# Supercritical Carbon Dioxide Extraction With Dimethyl Carbonate and Bio‐Ethanol as Co‐Solvents for the Determination of Pesticide Residues in Black Pepper by Low‐Pressure Gas Chromatography–Triple Quadrupole Mass Spectrometry

**DOI:** 10.1002/jssc.70368

**Published:** 2026-02-08

**Authors:** Alessia Arena, Mariosimone Zoccali, Luigi Mondello

**Affiliations:** ^1^ Chromaleont S.R.L., c/o Department of Chemical, Biological, Pharmaceutical and Environmental Sciences University of Messina Messina Italy; ^2^ Department of Mathematical and Computer Science, Physical Sciences and Earth Sciences University of Messina Messina Italy; ^3^ Messina Institute of Technology c/o Department of Chemical, Biological, Pharmaceutical and Environmental Sciences University of Messina Messina Italy

**Keywords:** black pepper, green solvents, pesticides, *Piper nigrum*, supercritical fluid extraction

## Abstract

The determination of pesticide residues in spices is analytically challenging due to their high content of essential oils and secondary metabolites that could interfere with detection and quantification. In this study, supercritical fluid extraction (SFE), using green co‐solvents, was evaluated for the extraction of 44 pesticides from black pepper (*Piper nigrum*). The extraction performance of bio‐ethanol (bio‐EtOH) and dimethyl carbonate (DMC) was compared under mild operating conditions. Optimal recoveries were obtained with 10% DMC, yielding an average of 92.6% across 44 pesticides, comparable to 93.7% using bio‐EtOH. Method performance was assessed by low‐pressure gas chromatography–triple quadrupole mass spectrometry (LP‐GC‐QqQMS). Linearity was achieved over the tested range, with limits of detection (within the range 0.1–17.9 µg kg^−1^) and quantification (within the range 0.3–59.8 µg kg^−1^) always below European Union (EU) maximum residue levels (MRLs). Accuracy ranged from 74.4% to 107.7%, with intra‐day precision between 0.8% and 16.1%. A pronounced matrix effect was observed (average ionization suppression: −47.6%). The application of the validated method to commercial black pepper samples revealed the presence of diphenylamine, quinalphos, and phosalone, with only quinalphos exceeding EU MRLs. These findings demonstrate that SFE with DMC as a co‐solvent provides an efficient, greener, and reliable extraction approach for multi‐residue pesticide analysis in a complex spice matrix. The combination of SFE with LP‐GC‐QqQMS supports the implementation of greener and high‐throughput monitoring workflows for complex spice matrices.

## 1 Introduction

Black pepper (Piper nigrum L.) is the oldest and most widely consumed spice worldwide, with a global market size valued at $4.7 billion in 2025 [1]. In addition to its culinary role as a widely appreciated food seasoning, black pepper is rich in bioactive compounds, primarily represented by piperine. This alkaloid contributes to its characteristic pungent flavor and exhibits potential anti‐inflammatory, antioxidant, anticancer, antihypertensive, antidepressant, and analgesic therapeutic properties [2, 3, 4].

P. nigrum crops are highly susceptible to pests and fungal infections, and are therefore frequently treated with various pesticides during cultivation and storage, to enhance productivity and reduce yield losses. In the European Union (EU) their application is strictly controlled by the maximum residue levels (MRLs) [5], to guarantee their sustainable use and to avoid health and environmental hazards, according to the Good Agricultural Practices recommendations [6].

Spices such as black pepper, along with hops, cocoa beans, coffee, and tea, have been classified as a “difficult or unique” commodity by the SANTE/11312/2021 v2 guidelines [7]. This is related to the large amount of essential oils and secondary metabolites (flavonoids, terpenes, and alkaloids), which may interfere with target analytes detection and quantification, making the extraction step a critical part of the analytical workflow. Among the conventional methods, the QuEChERS (Quick, Easy, Cheap, Effective, Rugged, and Safe) procedure, which couples an acetonitrile (ACN) extraction and a dispersive solid‐phase clean‐up with various types of sorbents (primary secondary amine, C18, or graphitized carbon black), is conventionally applied for pesticide analysis in black pepper [8, 9].

Supercritical fluid extraction (SFE) has gained growing interest as a viable alternative to conventional solvent‐based extraction methods. The efficiency of a SFE method is significantly influenced by several parameters, including temperature, pressure, co‐solvent type and amount, and extraction time. Optimizing these conditions is crucial to achieve reliable recovery in challenging matrices where co‐extracted compounds may interfere with the analytical performance.

The SFE technique has been employed for the extraction of pesticides in several matrices [10, 11, 12, 13], limiting its use to a small number of pesticides (< 10). Recently, Arena et al. analyzed apples exploiting the SFE performance towards an extended number of target pesticides (> 60), in addition to the use of green co‐solvents, namely bio‐ethanol (bio‐EtOH) and dimethyl carbonate (DMC), [14]. The best recovery values, in the 88.9%–116.9% range, with an average value of 106.4%, were obtained using bio‐EtOH. Slightly lower recovery values, from 45.3% to 111.1%, were obtained using DMC. Overall, the method achieved an average extraction yield of 85%, also providing cleaner extracts with a significantly lower amount of co‐extracted matrix components.

This study represents the first systematic assessment of DMC‐assisted SFE for multi‐residue pesticide extraction in black pepper, one of the most challenging spice matrices.

## 2 Materials and Methods

### 2.1 Chemicals

The 44 GC‐amenable pesticide standards (listed in Table 1), triphenyl phosphate (TPP, used as internal standard), bio‐EtOH (grade ≥ 99.5%) obtained by renewable feedstocks, and DMC (grade ≥ 99%) were purchased from Merck Life Science (Merck KGaA, Darmstadt, Germany). Carbon dioxide (CO_2_) was 99.998% of purity grade.

**TABLE 1:** List of the analyzed compounds along with molecular weights (MW), log *k_ow_*, recovery (measured at the best extraction conditions of each solvent), LoD and LoQ values (expressed as µg L^−1^), accuracy (evaluated at 50 µg L^−1^), precision (expressed as CV%), and matrix effect values.

			Recovery%						
Compound	MW (Da)	Log *k_ow_*	Bio‐EtOH	DMC	LoD	LoQ	Accuracy (%)	Precision (CV%)	Matrix effect (%)
Diphenylamine	169.2	3.5	89.9	90.8	1.9	6.2	102.4	7.0	−38
Chlorpropham	213.7	3.5	92.9	99.3	6.6	22.0	107.7	1.1	−41
Pencycuron	328.8	4.8	95.9	100.0	16.9	56.5	< LoQ	NA	−76
Terbufos	288.4	4.5	87.0	88.9	2.7	9.1	98.2	2.0	−93
Propyzamide	256.1	3.2	103.5	94.1	0.1	0.3	104.0	5.3	−49
Pyrimethanil	199.3	2.9	85.1	89.0	9.7	32.5	88.7	3.9	−28
Pirimicarb	238.3	1.7	87.6	88.1	11.0	36.7	86.9	6.1	−32
Vinclozolin	286.1	3.1	87.6	91.7	12.5	41.8	90.2	8.5	−33
Fenchlorphos	321.5	5.1	101.2	95.4	10.3	34.5	97.1	5.9	−42
Dimethoate	229.3	0.8	104.7	97.7	0.6	2.1	100.6	9.1	−44
Fenpropidin	273.5	5.5	97.0	90.1	6.5	21.6	92.5	3.5	−35
Dichlorobenzophenone	251.1	4.4	95.5	95.6	3.0	8.1	85.7	1.0	−55
Diethofencarb	267.3	2.8	97.3	93.1	6.3	21.1	83.0	3.8	−55
Tetraconazole	532.0	4.4	101.9	92.9	6.0	20.0	82.2	11.6	−44
Cyprodinil	225.3	3.1	89.4	87.4	7.9	26.5	82.6	8.9	−31
Pendimethalin	281.3	5.2	105.6	90.2	2.2	7.3	95.4	7.7	−46
Penconazole	284.2	4.4	97.7	90.5	7.3	24.2	79.8	10.0	−42
Quinalphos	298.3	4.4	102.2	92.4	11.5	38.3	80.6	8.5	−49
Fluopyram	396.8	4.5	96.0	91.1	10.9	36.2	81.9	3.9	−54
Procymidone	284.1	3.0	88.7	94.0	5.6	18.7	86.1	5.6	−50
Methidathion	302.3	2.4	88.6	88.1	5.5	18.5	83.3	6.6	−64
Flutriafol	223.3	3.3	84.1	87.1	8.2	27.4	82.5	9.6	−66
Mepanipyrim	301.0	2.3	88.0	88.7	10.0	33.3	87.0	3.7	−55
Myclobutanil	288.8	2.9	95.5	98.2	4.6	15.5	87.1	5.8	−43
Buprofezin	305.4	3.8	89.4	92.2	9.6	32.0	94.9	15.2	−46
Flusilazole	315.4	3.7	89.1	92.9	3.4	11.2	86.0	5.3	−41
Cyproconazole	291.8	2.9	90.6	97.0	7.2	23.9	81.1	6.7	−39
Bupirimate	316.4	2.7	94.2	96.8	3.1	10.2	85.3	4.9	−51
Kresoxim‐methyl	313.3	4.1	93.8	93.2	10.7	35.6	82.7	6.2	−40
Ancymidol	256.3	1.6	90.1	97.9	1.9	6.3	81.4	13.9	−40
Cyflufenamid	412.4	5.0	89.5	93.0	3.3	11.0	82.2	3.2	−46
Diniconazole	326.2	4.2	97.9	94.7	5.2	17.3	81.2	8.7	−49
Oxadixyl	278.3	1.8	91.9	89.6	4.7	15.6	78.2	0.9	−45
Triazophos	313.3	3.5	88.1	88.1	11.1	36.8	86.0	14.0	−59
Quinoxyfen	308.1	5.1	96.3	93.0	11.9	39.6	82.1	6.0	−37
Proquinazid	372.2	3.7	89.1	95.6	2.3	7.8	81.7	0.8	−37
Bromopropylate	428.1	4.8	82.6	88.4	10.2	33.9	74.4	7.8	−41
Bifenazate	300.4	4.2	80.3	95.6	5.2	17.3	82.4	12.1	−61
Bifenthrin	422.9	6.0	93.1	92.9	6.3	21.1	83.5	6.1	−48
Fenpropathrin	349.4	5.7	97.0	92.1	3.8	12.5	82.6	4.4	−46
Fenazaquin	306.4	5.7	101.2	95.6	11.4	37.9	98.2	9.5	−38
Fenamidone	311.4	4.1	91.0	90.6	7.0	23.5	81.2	16.1	−37
Tebufenpyrad	333.9	4.5	102.2	91.0	11.0	36.6	77.5	5.5	−40
Phosalone	367.8	4.4	110.8	89.7	17.9	59.8	< LoQ	NA	−89

The pesticide stock solutions were prepared at a concentration of 10000 mg L^−1^. The pesticide stock solutions were combined to obtain a solution containing the 44 pesticides at a concentration of 100 mg L^−1^ and stored in dark glass vials at 4°C until use.

Calibration standard solutions were prepared in DMC (solvent calibration) at the following concentration levels: 10, 50, 100, 250, 440, and 880 µg L^−1^, and in a blank pepper sample (matrix‐matched calibration) at the same concentration levels.

Percentage recovery values were calculated by comparing the peak areas (normalized for the IS) relative to a pre‐extraction spiked pepper with the values obtained from a post‐extraction spike.

### 2.2 Samples and Sample Preparation

Ten commercial samples of black peppercorns were purchased in markets located in Messina (Italy). The peppercorns were ground in a mortar and the obtained powder was spiked at a concentration of 20 mg kg^−1^. The mixture was kept for 3 h under magnetic agitation to ensure pesticide incorporation and solvent evaporation. The 0.2 mL stainless steel extraction vessels were directly filled with 100 mg of sample and placed into the SFE rack changer. Each sample was extracted in triplicate (*n* = 3).

### 2.3 Instrumentation

#### 2.3.1 SFE System

The employed Nexera‐UC off‐line SFE pre‐treatment system (Shimadzu, Kyoto, Japan) was equipped with: a CBM‐40 controller, an SFE‐30A auto‐extractor, an LC‐30AD_SF_ CO_2_ pump, an LC‐40AD_XR_ dual‐plunger parallel‐flow pumps, an LC‐40D make‐up pump, an SFC‐30A back pressure regulator (BPR), a DGU‐40 degasser, an FRC‐40 fraction collector.

Using DMC as a modifier, the SFE was carried out as follows: 1 min static extraction, 10% DMC; 10 min dynamic extraction, 10% DMC; flow rate: 1 mL min^−1^; BPR: 150 bar; vessel temperature: 30°C; make‐up solvent (DMC) flow rate: 0.9 mL min^−1^.

Using bio‐EtOH, the following SFE program was applied: 1 min static extraction 30% bio‐EtOH; 10 min dynamic extraction 30% bio‐EtOH; flow rate: 1 mL min^−1^; BPR: 150 bar; vessel temperature: 30°C; make‐up solvent (bio‐EtOH) flow rate: 0.7 mL min^−1^.

At the end of the dynamic extraction, the system was washed for 3 min using 100% of modifier at a flow rate of 1 mL min^−1^. After CO_2_ elimination, the liquid fraction was collected into glass vials and analyzed by low‐pressure gas chromatography–triple quadrupole mass spectrometry (LP‐GC‐QqQMS) (after a concentration step). The analysis was controlled using Shimadzu LabSolutions version 5.127 software.

#### 2.3.2 Low‐Pressure Gas Chromatography–Triple Quadrupole Mass Spectrometry System

The LP‐GC‐QqQMS analyses were performed using a GC‐2010 Plus system Shimadzu, coupled with a triple quadrupole mass spectrometer (TQ8040, Shimadzu). Injection was performed using an AOC‐20i autosampler and a split/splitless injector (injection temperature 280°C) equipped with a focus liner (volume: 810 µL). The injection was performed in splitless mode (3 µL) using the high‐pressure injection mode function (250 kPa for 0.5 min).

An uncoated capillary pre‐column (0.48 m × 0.1 mm ID) was connected, by using a SilTite μ‐Union (Trajan Scientific Australia Pty Ltd.), to a non‐polar Equity‐5 (poly[5 % diphenyl/95 % dimethyl siloxane]) column of the following dimension: 5 m × 0.53 mm ID × 0.53 µm *d_f_*. Both columns were provided by Merck KGaA.

Separation was achieved by holding the GC oven for 2 min at 40°C, followed by a temperature increase of 30°C min^−1^ up to 320°C (total run time 11.33 min). Helium was used as carrier gas with an initial pressure of 35.6 kPa and an average linear velocity mode of 100 cm s^−1^ into the analytical column (working under constant linear velocity mode). QqQMS conditions: electron ionization (70 eV); interface and ion source temperatures: 320°C and 280°C, respectively. Argon was used as collision gas at 200 kPa. Acquisition modes: SCAN (using a 50–600 *m/z* mass range and a 3.3 Hz spectral production frequency) and multiple reaction monitoring (MRM). The GCMS Solution v.4.45 software (Shimadzu) was used for data collection and processing.

**FIGURE 1:**
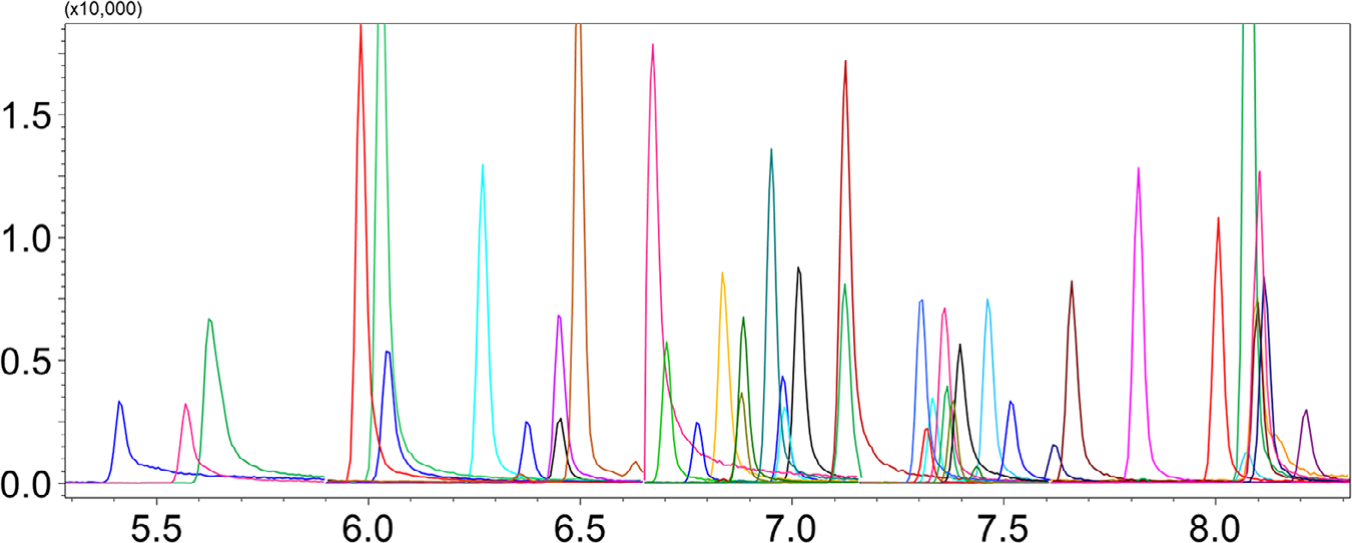
LP‐GC‐QqQMS chromatogram, acquired in MRM mode, of a spiked black pepper sample (at a concentration level of 20 mg kg^−1^).

Figure 1 shows the LP‐GC‐QqQMS chromatogram of a spiked black pepper sample (at a concentration level of 20 mg kg^−1^). Refer to Table S1 for compounds retention time, quantifier and qualifier MRM transitions, and collision energies (CEs).

## 3 Results and Discussion

### 3.1 SFE Optimization

The research aim was to extend the SFE applicability to the analysis of a high number of pesticides (compared to literature data) in black pepper, recognized as a challenging matrix by the SANTE guidelines [7].

**FIGURE 2:**
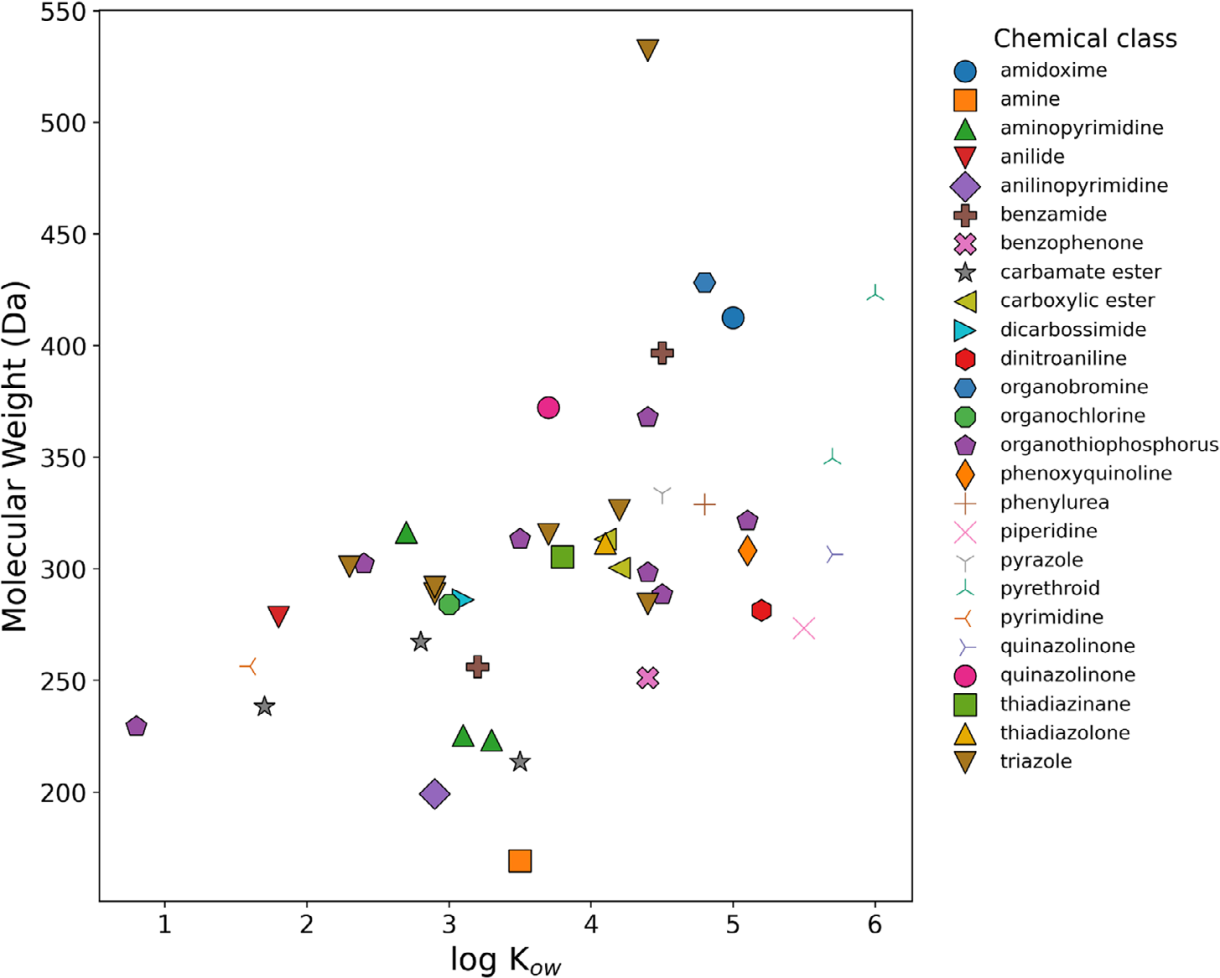
Scatter plot of the investigated pesticides molecular weight and log *k_ow_*.

The investigated molecules were characterized by a wide range of polarity (with log *k_ow_* spanning from 0.8 of dimethoate to 6.0 of bifenthrin) and molecular weight (across the 162–532 Da range) (Table 1). Additionally, 25 different chemical classes were covered, mainly represented by seven organothiophosphorus, seven triazoles, three carbamate esters, three aminopyrimidines, two carboxylic esters, two benzamides, and two pyrethroids. A scatter plot showing the relationship between pesticides' log *k_ow_* (*x*‐axis) and their molecular weight (*y*‐axis) is reported in Figure 2, with each chemical class represented by different color and shape dots.

**FIGURE 3:**
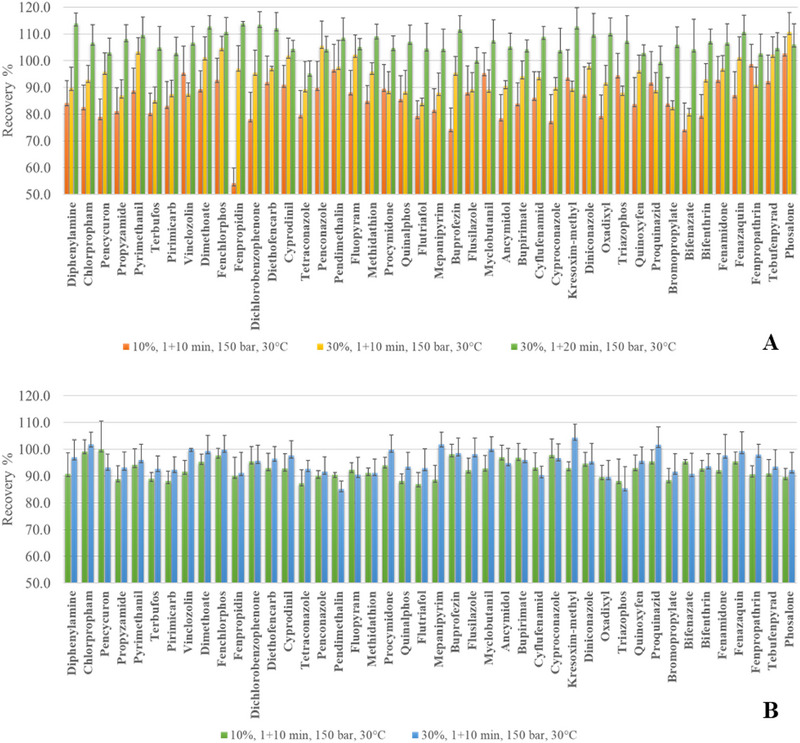
Bar graphs showing pesticide recoveries obtained using the tested extraction methods for bio‐EtOH (A) and DMC (B).

The initial SFE conditions were based on a previously published study [14], namely a total flow rate of 1 mL min^−^^1^ and a pressure of 150 bar (BPR). To broaden the applicability of the method to polar compounds, the addition of a polar modifier was found to be essential. In this context, the extraction performance of two green co‐solvents, namely bio‐EtOH and DMC, was evaluated. The co‐solvent content was tested at 10% and 30%. Using 30% bio‐EtOH, an average increase in the pesticide recoveries of 10.2% was observed. An improvement greater than 15% was obtained for 22 compounds, while a substantial enhancement was obtained for fenpropidin, whose recovery increased from 54.2% to 97.0%. Overall, the recovery values ranged from 80.3% (bifenazate) to 110.8% (phosalone), with an average of 93.7%. A further improvement (average 106.8%) was achieved by extending the dynamic extraction step from 10 to 20 min. However, this yield enhancement was considered negligible and did not justify the additional 10 min extraction time. Comparable results were obtained using DMC. Increasing the modifier content from 10% to 30% led to an average recovery improvement of 4.4%, which was not deemed significant. Therefore, the 10% DMC amount was selected, yielding recoveries ranging from 87.1% (flutriafol) to 100.0% (pencycuron), with an average of 92.6%. Each extraction was performed in triplicate, and the coefficient of variation (CV%) was always below 12%. The bar charts (along with error bars) in Figure 3 illustrate and compare the recoveries of the 44 analyzed pesticides obtained under the tested extraction conditions. Three replicates were performed.

The yields obtained employing the best extraction conditions for both solvents are also reported in Table 1.

The extraction was performed at 30°C using 1 min static followed by 10 min of dynamic extraction with 10% DMC. Therefore, compared with [14], a reduced co‐solvent amount was employed, in addition to milder conditions, namely 30°C and 10 min of dynamic extraction time. This could be ascribed to the large surface area of finely ground black pepper, allowing a deeper penetration of the sc‐CO_2_ fluid into the matrix and thus improving the overall extraction yields.

Directly comparing the developed method with the literature‐reported QuEChERS‐based ones [8, 9], SFE provides comparable results, in terms of sample preparation throughput, with a total extraction time of 14 min (also considering instrument wash), extraction efficiency, and solvent consumption (10 mL of ACN vs. 14 mL of DMC). However, the use of a greener solvent ensured compliance with the Green Analytical Chemistry and Green Sample Preparation principles [15]. Despite the use of a sophisticated apparatus, the degree of automation was improved, allowing a reduction of manual sample preparation steps and operator exposure to chemical solvents.

In addition to the demonstrated SFE performance using DMC as co‐solvent, the high optimal linear velocity achievable in LP‐GC under sub‑atmospheric pressure conditions speeds up the analysis time by approximately threefold (given that conventional GC methods typically require around 30 min [16, 17]), with the last compound eluting at 8.2 min. Furthermore, the high sample capacity allows the injection of larger sample volumes without overloading, thereby improving analyte detectability.

### 3.2 Figures of Merit

The SFE//LP‐GC‐QqQMS method was evaluated in terms of: linearity within the calibration range, limits of detection (LoD) and quantification (LoQ), accuracy, precision, and matrix effect (ME). The obtained results are summarized in Table 1. Table S1 reports calibration ranges, matrix‐matched calibration equations, along with MRLs values. The SFE extracts have been concentrated under a gentle stream of nitrogen and injected into the LP‐GC‐QqQMS system without any additional steps (such as derivatization).

Matrix‐matched calibration curves were established using a black pepper sample (with no detectable pesticides) spiked at six concentration levels and treated following the developed SFE procedure using DMC as co‐solvent. The LoD and LoQ values were calculated by multiplying the standard deviation of the analyte area, relative to the black pepper fortified at the lowest concentration level, three and 10 times, respectively, and then by dividing the result by the slope of the calibration curve. The LoD values ranged from 0.1 µg kg^−1^ for propyzamide to 17.9 µg kg^−1^ for phosalone, whereas LoQ values ranged from 0.3 to 59.8 µg kg^−1^ for the same pesticides. As can be seen in Table 1, the LoQ values were always below the EU MRLs related to peppercorn [6]. The lowest point of calibration was either equal (for 13 pesticides) or below the MRLs.

Accuracy (*n *= 3) was measured by analyzing a spiked black pepper sample at the 50 µg kg^−1^ concentration level. The obtained values ranged between 74.4% for bromopropylate and 107.7% for chlorpropham, with an average value of 86.9% (for pencycuron and phosalone, the accuracy level was lower than their LoQ). Intra‐day precision (*n* = 6) was calculated by analyzing a spiked black pepper sample at the same concentration level, with CV% values ranging from 0.8 (proquinazid) to 16.1% (fenamidone) (average = 6.8%).

ME values were calculated as the difference between the slope of the post‐extraction calibration curve and the solvent‐only (SO) calibration curve, divided by the slope of the SO calibration curve; the derived value was then expressed as a percentage. For all the analyzed compounds, a signal suppression was observed, with %ME values ranging from −93% (for terbufos) to −28% (for pyrimethanil), with an average value of −47.6%. Considering the absence of an extract clean‑up step, this signal reduction is attributed to the intense elution of co‑extracted matrix components, which leads to reduced ionization efficiency and/or a less efficient transfer of analyte ions to the MS analyzer [18]. However, despite the observed significant ion suppression, quantification reliability was preserved through matrix‐matched calibration.

**FIGURE 4:**
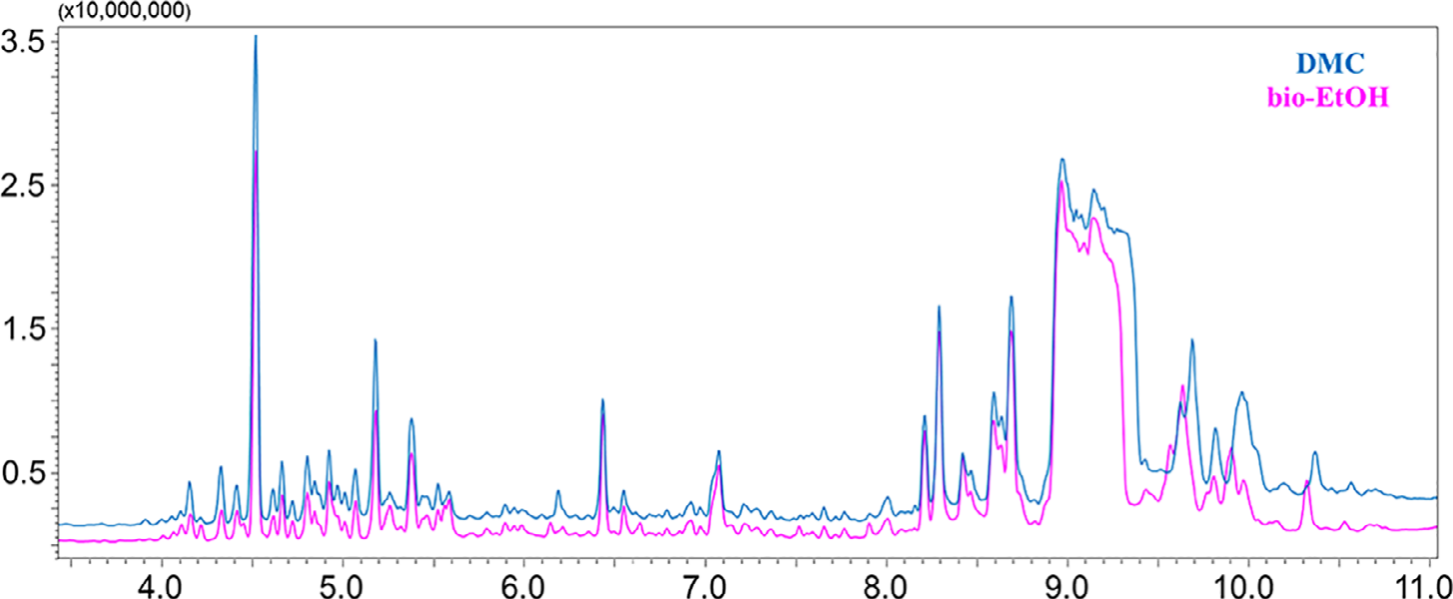
Comparison of the total ion current chromatograms of the DMC (upper trace) and bio‐EtOH (lower trace) black pepper extracts.

As previously reported [14], extraction selectivity was assessed, and Figure 4 shows that the two extracts are highly similar in both the amount and type of co‐extracted matrix components. Thus, considering the volatile fraction of the extract, no appreciable differences were observed.

### 3.3 Analysis of Commercial Samples

Ten black pepper samples, collected from local markets (Messina, Italy), were analyzed using the developed method using DMC as SFE co‐solvent and injected in the LP‐GC‐QqQMS system after a concentration step. Three replicates were performed for each sample.

Diphenylamine was detected in three samples in the range 6.3–11.3 µg kg^−1^ (always below the EU MRL of 0.05 µg kg^−1^). Quinalphos was detected in three samples in the range 9.5–52.6 µg kg^−1^, only in one case above the EU MRL of 0.05 µg kg^−1^. Finally, phosalone was detected in two samples, at a concentration of 122.8 and 167.9 µg kg^−1^ (always below the EU MRL of 2 mg kg^−1^).

## 4 Conclusions

This work demonstrates the applicability of supercritical CO_2_ extraction combined with green co‐solvents for the determination of pesticide residues in black pepper. Among the tested conditions, 10% DMC yielded optimal recoveries and method performance, while enabling operation under milder temperature (30°C) and pressure (150 bar) conditions compared with a previously reported approach. The validated SFE//LP‐GC‐QqQMS method provided adequate analyte detectability, precision, and accuracy, with LoQs always below EU MRLs. Analysis of commercial samples confirmed its suitability for real‐world applications, highlighting occasional non‐compliance with regulatory thresholds. The proposed approach represents a greener and more effective alternative to conventional solvent‑based methods for pesticide analysis in a complex spice matrix such as black pepper. Future studies should further investigate MEs and evaluate the scalability of this method to other challenging food commodities, strengthening its role in routine pesticide monitoring and food safety assurance.

## Author Contributions

**Alessia Arena**: conceptualization, methodology, formal analysis, investigation, writing – original draft, writing – review and editing. **Mariosimone Zoccali**: conceptualization, methodology, writing – original draft, writing – review and editing, supervision. **Luigi Mondello**: conceptualization, writing – review and editing, funding acquisition, resources, supervision.

## Conflicts of Interest

The authors declare no conflicts of interest.

## Supporting information




**Supporting File 1**: jssc70368‐sup‐0001‐SuppMat.docx.

## Data Availability

The data supporting this study's findings are available from the corresponding author upon reasonable request.
