# Supplementary material for: Supercritical Carbon Dioxide Extraction With Dimethyl Carbonate and Bio‐Ethanol as Co‐Solvents for the Determination of Pesticide Residues in Black Pepper by Low‐Pressure Gas Chromatography–Triple Quadrupole Mass Spectrometry
Source: J Sep Sci. 2026 Feb 8;49(2):e70368. doi: 10.1002/jssc.70368 (PMC12883146; doi:10.1002/jssc.70368)
Supplement: Supplementary file 1 — Supporting File 1: jssc70368‐sup‐0001‐SuppMat.docx. [file JSSC-49-e70368-s001.docx]

**Supercritical carbon dioxide extraction of pesticides residues from black pepper (*Piper nigrum*) using dimethyl carbonate and bio-ethanol co-solvents coupled to**

**low-pressure gas chromatography–triple quadrupole mass spectrometry**

Alessia Arena^1^, Mariosimone Zoccali^2,*^, Luigi Mondello^1,3^

*^1^ Chromaleont s.r.l., c/o Department of Chemical, Biological, Pharmaceutical and Environmental Sciences, University of Messina, Viale G. Palatucci 13, 98168, Messina, Italy*

*^2^ Department of Mathematical and Computer Science, Physical Sciences and Earth Sciences, University of Messina, Viale F. Stagno d'Alcontres 31, 98168, Messina, Italy*

*^3^ Messina Institute of Technology c/o Department of Chemical, Biological, Pharmaceutical and Environmental Sciences, University of Messina, Viale G. Palatucci 13, 98168, Messina, Italy*

*Corresponding author.

Tel. 0039-090-6765465

*E-mail address*: mzoccali@unime.it

**Table S1.** List of analysed pesticides, along with retention time, MRLs values, quantifier and qualifier transitions [with collision energies (eV)], calibration range, and matrix-matched calibration equation.

| **Compounds** | **RT (min)** | **MRL**  **(mg kg^-1^)** | **Quantifier transition (CE)** | **Qualifier transition (CE)** | **Calibration range**  **(µg kg^-1^)** | **Calibration equation** |
| --- | --- | --- | --- | --- | --- | --- |
| Diphenylamine | 5.5 | 0.05 | 169>66 (24) | 169>77 (28) | 10-880 | Y=0.0074X+0.0296 |
| Chlorpropham | 5.6 | 0.05 | 213>171 (6) | 213>127 (14) | 10-880 | Y=0.007X+0.0003 |
| Pencycuron | 5.7 | 0.10 | 180>125 (9) | 125>89 (15) | 100-880 | Y=0.0074X+0.2092 |
| Terbufos | 6.0 | 0.01 | 231>129 (26) | 231>175 (14) | 10-880 | Y=0.031X+0.1035 |
| Propyzamide | 6.0 | 0.05 | 173>145 (16) | 173>74 (28) | 10-880 | Y=0.0333X+2.5125 |
| Pyrimethanil | 6.0 | 0.05 | 198>183 (14) | 198>158 (18) | 50-880 | Y=0.0072X+0.094 |
| Pirimicarb | 6.3 | 0.05 | 238>166 (12) | 238>72 (24) | 10-880 | Y=0.0216X+0.4097 |
| Vinclozolin | 6.4 | 0.05 | 285>212 (12) | 285>178 (14) | 10-880 | Y=0.0041X+0.0725 |
| Fenchlorphos | 6.5 | 0.10 | 285>270 (16) | 287>272 (18) | 10-880 | Y=0.0102X+0.052 |
| Dimethoate | 6.5 | 0.05 | 125>79 (8) | 125>47 (14) | 10-880 | Y=0.0046X+0.0635 |
| Fenpropidin | 6.5 | 0.05 | 98>55 (15) | 273>98 (21) | 50-880 | Y=0.0017X+0.0873 |
| Dichlorobenzophenone | 6.7 | NR | 139>111 (14) | 139>75 (20) | 10-880 | Y=0.007X+0.0747 |
| Diethofencarb | 6.7 | 0.05 | 267>225 (8) | 267>196 (14) | 50-880 | Y=0.0058X+0.2296 |
| Tetraconazole | 6.8 | 0.05 | 336>218 (14) | 336>204 (28) | 50-880 | Y=0.0031X+0.1072 |
| Cyprodinil | 6.8 | 0.10 | 224>208 (16) | 224>131 (14) | 10-880 | Y=0.0145X+0.5846 |
| Pendimethalin | 6.9 | 0.05 | 252>162 (10) | 252>191 (8) | 10-880 | Y=0.0044X+0.0949 |
| Penconazole | 6.9 | 0.05 | 248>192 (14) | 248>157 (26) | 10-880 | Y=0.0087X+0.4266 |
| Quinalphos | 7.0 | 0.05 | 146>118 (10) | 157>129 (14) | 50-880 | Y=0.0157X+0.7956 |
| Fluopyram | 7.0 | 0.05 | 173>145 (30) | 173>95 (30) | 50-880 | Y=0.0048X+0.2791 |
| Procymidone | 7.0 | 0.05 | 283>96 (10) | 283>255 (12) | 50-880 | Y=0.0035X+0.1516 |
| Methidathion | 7.0 | 0.10 | 145>85 (8) | 145>58 (14) | 10-880 | Y=0.0083X+0.2821 |
| Flutriafol | 7.1 | 0.05 | 219>123 (14) | 219>95 (28) | 50-880 | Y=0.0069X+0.385 |
| Mepanipyrim | 7.1 | 0.05 | 223>222 (10) | 222>221 (6) | 10-880 | Y=0.0225X+0.7915 |
| Myclobutanil | 7.3 | 0.05 | 179>125 (14) | 179>152 (8) | 10-880 | Y=0.0112X+0.5056 |
| Buprofezin | 7.3 | 0.05 | 172>57 (14) | 172>131 (6) | 10-880 | Y=0.0025X+0.3004 |
| Flusilazole | 7.3 | 0.05 | 233>165 (14) | 233>152 (14) | 10-880 | Y=0.0047X+0.2144 |
| Cyproconazole | 7.4 | 0.05 | 222>125 (24) | 222>82 (12) | 10-880 | Y=0.0112X+0.4768 |
| Bupirimate | 7.4 | 0.05 | 273>193 (8) | 273>150 (8) | 10-880 | Y=0.0039X+0.1454 |
| Kresoxim-methyl | 7.4 | 0.05 | 206>116 (6) | 206>131 (14) | 10-880 | Y=0.004X+0.1801 |
| Ancymidol | 7.4 | NR | 228>121 (15) | 121>78 (25) | 10-880 | Y=0.01X+0.3572 |
| Cyflufenamid | 7.4 | 0.05 | 412>295 (8) | 412>118 (26) | 50-880 | Y=0.0007X+0.0559 |
| Diniconazole | 7.5 | 0.05 | 268>232 (12) | 268>149 (24) | 10-880 | Y=0.0095X+0.4104 |
| Oxadixyl | 7.5 | 0.02 | 163>132 (8) | 163>117 (24) | 10-880 | Y=0.0053X+0.1925 |
| Triazophos | 7.6 | 0.07 | 257>162 (8) | 291>81 (24) | 50-880 | Y=0.0018X+0.0871 |
| Quinoxyfen | 7.7 | 0.05 | 237>208 (28) | 237>182 (28) | 10-880 | Y=0.0129X+0.5509 |
| Proquinazid | 7.8 | 0.05 | 288>245 (18) | 330>288 (12) | 10-880 | Y=0.0183X+0.6483 |
| Bromopropylate | 8.0 | 0.05 | 341>183 (18) | 341>185 (20) | 10-880 | Y=0.0069X+0.3253 |
| Bifenazate | 8.1 | 0.05 | 300>258 (8) | 300>199 (20) | 50-880 | Y=0.0015X+0.1372 |
| Bifenthrin | 8.1 | 0.03 | 181>166 (12) | 181>153 (8) | 10-880 | Y=0.0493X+1.7949 |
| Fenpropathrin | 8.1 | 0.02 | 265>210 (12) | 265>172 (14) | 10-880 | Y=0.0029X+0.12 |
| Fenazaquin | 8.1 | 0.05 | 160>145 (8) | 145>115 (24) | 10-880 | Y=0.0207X+1.2941 |
| Fenamidone | 8.1 | 0.05 | 268>180 (16) | 268>77 (28) | 10-880 | Y=0.0017X+0.2168 |
| Tebufenpyrad | 8.1 | 0.05 | 333>171 (20) | 333>276 (8) | 10-880 | Y=0.0056X+0.3099 |
| Phosalone | 8.2 | 2.00 | 182>111 (14) | 182>138 (8) | 100-880 | Y=0.0009X+0.1486 |
